# Valorization of Fibrous Plant-Based Food Waste as Biosorbents for Remediation of Heavy Metals from Wastewater—A Review

**DOI:** 10.3390/molecules28104205

**Published:** 2023-05-20

**Authors:** Ahasanul Karim, Zarifeh Raji, Antoine Karam, Seddik Khalloufi

**Affiliations:** 1 Department of Soils and Agri-Food Engineering, Université Laval, Quebec, QC G1V 0A6, Canada; md-ahasanul.karim.1@ulaval.ca (A.K.);; 2 Institute of Nutrition and Functional Foods (INAF), Université Laval, Quebec, QC G1V 0A6, Canada

**Keywords:** biosorbents, food-waste valorization, plant fibers, heavy metals, wastewater treatment

## Abstract

Mobilization of heavy metals in the environment has been a matter of concern for several decades due to their toxicity for humans, environments, and other living organisms. In recent years, use of inexpensive and abundantly available biosorbents generated from fibrous plant-based food-waste materials to remove heavy metals has garnered considerable research attention. The aim of this review is to investigate the applicability of using fibrous plant-based food waste, which comprises different components such as pectin, hemicellulose, cellulose, and lignin, to remove heavy metals from wastewater. This contribution confirms that plant-fiber-based food waste has the potential to bind heavy metals from wastewater and aqueous solutions. The binding capacities of these biosorbents vary depending on the source, chemical structure, type of metal, modification technology applied, and process conditions used to improve functionalities. This review concludes with a discussion of arguments and prospects, as well as future research directions, to support valorization of fibrous plant-based food waste as an efficient and promising strategy for water purification.

## 1. Introduction

“Heavy metals” are associated with environmental pollution, food contamination, and toxicity and have adverse effects on terrestrial and aquatic ecosystems and animal and human health [1,2]. Hazardous heavy metals and metalloids, such as arsenic (As), cadmium (Cd), chromium (Cr), lead (Pb), and mercury (Hg), and several essential heavy metals, such as copper (Cu), iron (Fe), manganese (Mn), nickel (Ni), and zinc (Zn), above threshold levels, have been identified as priority contaminants and one of the key environmental issues of global concern over the last several decades due to their mobility in terrestrial and natural aquatic ecosystems and their carcinogenic nature [3,4]. Municipal and industrial wastewaters frequently include a variety of heavy-metal ions, posing a serious threat to the aquatic ecosystem and environment [5]. This is because heavy metals are stable and persistent environmental pollutants due to their nonbiodegradability and high toxicity [6,7]. Furthermore, they possess a tendency to bioaccumulate and biomagnify through the food chain, causing serious threats to humans and other living organisms directly and indirectly [8]. Consequently, metal-polluted wastewater must be treated prior to discharge into the environment. Table 1 lists several common heavy metals, their sources, and associated health problems.

Several technologies have been employed for eliminating heavy metals from wastewater, contaminated aquatic media, and industrial effluents over the last three decades, including chemical precipitation [26], solvent extraction [27], coagulation–flocculation [28], advanced oxidation [29], membrane filtration [30], reverse osmosis [31], ion exchange [26], ozonation [32], photocatalysis [33], adsorption [34,35], biosorption/bioaccumulation [36], bioleaching [37], phytoextraction using hydroponic systems coupled with bioremediation [38], phytofiltration [39], electroremediation [34], etc. However, there is no single best method to provide adequate treatment, as each treatment has its own distinct benefits and shortcomings, not only in terms of cost but also in terms of consistency, efficacy, practicability, viability, and operational difficulties (Table 2) as well as environmental impact [40].

Among them, sorption of heavy metals from aqueous media is hailed as a promising and frequently employed technique due to its high removal efficiency for metal ions, even at trace concentrations, and ease of operation compared to conventional techniques [45]. However, use of sorption is limited due to the high cost and insufficient regeneration of frequently used adsorbents such as commercial activated carbon [34,35]. Both non-plant- and plant-based materials are employed as low-cost adsorbents. Zeolites, clay, chitosan, red mud, dairy sludge, and metal oxides are all used as adsorbents in non-plant-based materials [46]. Prospects for using plant-based waste as adsorbents, including industrial byproducts and agricultural waste, are deemed highly promising [47]. On the basis of the theories of a “circular bioeconomy” and “green chemistry”, transformation of agricultural waste and residues into products with added value is viewed as a cheap, renewable, abundantly accessible, and ecologically beneficial process [47].

Various fibrous plant-based food biomasses have been employed as precursors for production of adsorbents, such as plant leaves [48], lentil husks [49], agricultural peels [50], coconut biomass [51], etc. However, they need to be treated or modified before being used as adsorbents for metal ions. This is due to the fact that application of untreated plant waste may result in a number of problems, such as decreased sorption capacity, increased biological and chemical oxygen demand, and an increase in total organic carbon due to the discharge of soluble organic carbon remaining in the plant materials [52]. Thus, while application of biosorption for removal of hazardous metals using inexpensive raw materials has attracted substantial interest, various obstacles must be solved before these materials can be employed commercially. On the other hand, various food and agricultural waste are created globally and could be used as soil supplements to improve soil health and crop yield [53]. However, direct application of such waste may endanger soil health, particularly soil chemical and microbiological characteristics [54]. Bioconversion of agricultural and food waste into nonhazardous and stable soil additives is therefore a potential option. This would not only decrease the dangers connected with environmental burdens but also assure safe disposal and use of the end product as sustainable soil additives [55].

Food waste results in roughly 20 million tons (Mt) of CO_2_-equivalent GHG emissions annually [56]. According to a recent estimate by the Food and Agricultural Organization (FAO), the worldwide food-waste market is valued at over 750 billion USD annually [57]. Forty percent of domestic food output is wasted annually in the United States and Canada, amounting to 165 billion and 27 billion USD, respectively [58]. In this context, recycling or reusing fibrous plant-based food waste for developing affordable purification technology in the water, soil, and food industries could be an attractive component of circular bioeconomies as well as provide greater environmental benefits. Recently, plant fibers produced from agricultural waste have been characterized as excellent adsorbents for environmental remediation of effluents [59,60,61].

Plant fibers are found as structural elements in all higher plants [62]. Examples of plant fibers mainly include lignocellulose-based materials made of lignin, hemicellulose, and cellulose; when mixed with polyphenols, pectin, and proteins, they are utilized for sorption of trace metal ions [63] and dyes [64] as well as oil removal [65] from water. The components of fibers vary not only in physiological activity and chemical structure from one source to another but also in their capacity to bind essential elements such as Ca, Cu, Fe, and Zn [66] and heavy metals such as As, Cd, Hg, and Pb [67]. The performance of fibers depends on several factors: physicochemical parameters, functionality, and modification technology to improve functionality [68,69]. Fibers are found in plant-based foods such as nuts and seeds (beans, split peas, soybeans, corn, sunflowers, barley, oats, wheat, almonds, pumpkins, lentils, etc.), legumes or vegetables (cauliflower, carrots, broccoli, celery, cabbage, turnip greens, brussels sprouts, potatoes, artichokes, eggplants, beets, cauliflower, endives, turnips, fennel, onions, leeks, rutabagas, etc.), and fruits (guavas, mangoes, strawberries, pomegranates, bananas, prunes, apples, raspberries, pears, avocados, blackberries, oranges, pineapples, etc.). Thereby, plant-based food wastes contain plant fibers. Fiber is a blanket term that applies to any type of carbohydrate that humans cannot digest. In a characterization study of dietary fiber lignins from 11 fruits and vegetables using the DFRC method, Bunzel and Seiler [70] found that apples, kiwis, pears, asparagus, carrots, curly kale, kohlrabi, radishes, small radishes, rhubarb, and spinach contained 9.8, 11.9, 12.9, 18.0, 10.3, 33,4, 6.2, 12.6, 18.3, 26.7, and 28.5% insoluble fiber, respectively. Natural fibers of plant origin (plant fibers) can come from different parts of a plant.

The purpose of this review is to highlight potential applications and research in the field of biosorption, utilizing a variety of low-cost materials, most notably fibrous plant-based food waste, including their biomass parts and fiber components, for heavy-metal remediation from wastewater. Nevertheless, the influences of fiber structures and properties on the sorption process, the mechanisms of their actions, and the regeneration capabilities of fibrous plant-based food waste are reviewed. Finally, the main challenges and prospects for heavy-metal sorption using fibrous plant-based food waste in water or soil are highlighted for future research directions. This review specifies that it encompasses research published between 1997 and 2022 and that the search criteria included “fibrous plant-based food waste”, “biosorbent”, “plant fibers”, “heavy metal”, and “wastewater”.

## 2. Fibrous Plant-Based Food Waste for Sorbing Heavy Metals

### 2.1. Plant-Fiber Components

Plant fibers are found as structural elements in agricultural crops and in their botanical parts, such as nuts, grains, or seeds (beans, split peas, soybeans, corn, sunflowers, barley, oats, wheat, almonds, pumpkins, etc.); lentils, legumes, or vegetables (cauliflower, carrots, broccoli, celery, cabbage, turnip greens, brussels sprouts, potatoes, artichokes, eggplants, beets, cauliflower, endives, turnips, fennel, onions, leeks, rutabagas, etc.); and fruits (guavas, mangoes, strawberries, pomegranates, bananas, prunes, apples, raspberries, pears, avocados, blackberries, oranges, pineapples, etc.). Plant fibers have a complex structure that is really made up of a cell wall and a central lumen channel. The middle lamella, the primary wall, and the secondary wall are the three components that make up a cell wall [71]. The primary wall is made up of disorganized cellulose in a pectin, hemicellulose, and lignin matrix. The secondary wall is composed of crystalline cellulose and is separated into three sections: the exterior, middle, and interior secondary walls [72]. The chemical components of plant fibers, including cellulose, lignin, hemicellulose, pectin, and wax, can be different depending on their sources and origins [71]. In food science, cellulose, lignin, pectin, and hemicellulose derived or extracted from fibrous plant foods or contained in plant foods or plant-fiber matrices are designated as cellulose fiber, lignin fiber, pectin fiber, and hemicellulose fiber, respectively.

The main component of plant cells is generally cellulose, which is arranged in microfibrils and surrounded by hemicellulose, which includes xylans, mannans, glucomannans, galactans, and arabinogalactans as well as lignin, pectin, and trace amounts of protein [59,73]. Fibers include functional groups such as carboxyl, phenolic, lactonic, and hydroxyl groups that bind to metals and remove them from aqueous environments. These functional groups interact with metal ions and act as hydrogen-ion replacements. Over a wide pH range, the process includes electrostatic and dispersive interactions between cations and the acidic surface area [66]. Feng and Guo [74] showed how Pb^2+^, Cd^2+^, and Ni^2+^ ions were attached to modified orange peel by inclusion of carboxyl and hydroxyl groups. The constancy of metal-fiber complexes varies with the type of metal, the experimental settings, the fiber sources, and other factors, according to published studies [59,66]. Al-Ghouti and Li [75] revealed that raw date pits may be utilized to remove Cu^2+^ and Cd^2+^ through the processes of complexation, coordination, chelation, ion exchange, and adsorption.

### 2.2. Fibrous Plant-Based Biomass Parts

The different parts of fibrous plant-based biomasses, considered low-cost potential metal biosorbents, are leaves, stems, stalks, roots, bagasse, seeds, shells, peels, husks, bark, and fibers [46,76,77]. Various plant fiber-based biomasses have been widely used as natural materials, pretreated or chemically modified, for heavy-metal removal from aqueous media, including wastewater and aqueous solutions. These are carrot residue [78]; potato peel [79]; sunflower stalks and leaves [80]; coconut shells [81]; seed shells [82]; coffee husks [83]; sugar-beet pulp [84,85]; crude olive stones [86]; olive-oil waste and hydrolyzed olive cake [87,88]; apple peel beads [89]; citrus peels [90], the shells of hazelnuts and almonds [91]; physic seed hulls [92]; rice husks [93]; neem bark [94]; tea waste [95]; sunflower, potato, canola and walnut shells [80]; sugarcane bagasse [96]; bamboo charcoal [97]; pistachio-hull waste [98]; cashew-nut shells [99]; agave bagasse [100]; *Rosa damascena* leaf powder [101]; ajwa date pits [102]; chemically modified orange peel [74]; banana peel and chemically modified banana peel [103]; orange and potato immobilized on sodium alginate beads [104]; olive-oil waste and hydrolyzed olive cake [87,88]; banana thrunk fibers [105]; and cellulose fibers extracted from pineapple leaves [106] (Table 3).

### 2.3. Factors Influencing the Sorption Efficiencies of Fibrous Plants

The capacities of biowaste-derived sorbents for metal-ion sorption are, however, dependent on the physicochemical properties of the prepared sorbents. The most important properties of these sorbents are cation exchange capacity (CEC), pore distribution, porosity, specific surface area, surface functional groups, etc. [113,114]. Lyu and Wang [115] stated that larger specific surface areas led to higher metal (Cd^2+^, Cu^2+^, Pb^2+^, and Zn^2+^) sorption from their aqueous solutions using insoluble fiber from soybean dregs (okara). They described that the smaller particle size of insoluble okara fiber demonstrated a higher oil-holding capacity (OHC), CEC, and sorption capacity of heavy metals. In another study, ultramicro-grinding of insoluble fiber from carrot pomace decreased the particle size of the total fiber and increased its Brunauer–Emmett–Teller surface area from 0.374 to 1.835 m^2^/g, leading to an increase in the water-holding capacity (WHC), swelling capacity (SC), and OHC, as well as the nitrite- and Pb^2+^-ion-adsorbing capacities [116]. Furthermore, Al-Ghouti and Li [75] discovered that the volume of solute (Cu^2+^ and Cd^2+^) adsorbed increases as the particle size of the adsorbents decreases. The crystallinity of the cellulosic structure also affects sorption kinetics [117]. Amorphous regions have a positive correlation, while crystalline structures have a negative correlation with heavy-metal sorption [118]. With a rise in pH, the negative charge density of a fiber surface improves, which leads to an increase in sorption of heavy metals [119]. Higher Cd^2+^, Cu^2+^, and Pb^2+^ bind to biosorbents by having more acidic functional groups and negative zeta potential [120]. Similarly, Wang and Yang [121] observed that there is a positive correlation between efficiency of removing heavy metals and pH, and that removal efficiency improved when the pH was increased to 7.

**Figure 1:**
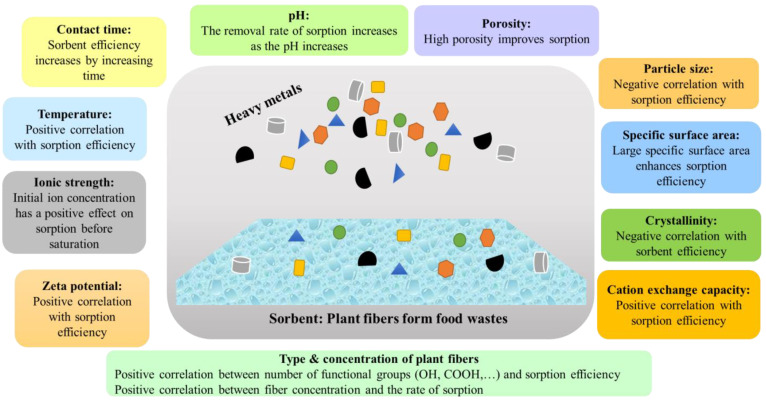
Critical factors affecting sorption efficiency of heavy metals from aqueous media.

Nevertheless, the biological origins of plants and types of processing have a considerable influence on sorption properties. For instance, beet pectin demonstrated a higher affinity for Pb^2+^ and Cu^2+^, while citrus pectin did so for Ni^2+^ and apple pectin did so for Co^2+^ [122]. Requena and González [123] showed that CEC significantly varies depending on the source of fiber; for example, CEC values of 3.5, 4.1, 2.6, 2.7, 2.6, and 1.3 meq/g were reported for ashen agave bagasse, green agave bagasse, cabuche, prickly pear peel, palm flowers, and the leaves of smooth amaranth, respectively. Nevertheless, sorption capacity largely depends on solution ion strength [124]. The higher charge density of Cu^2+^ (116 C mm^−3^) results in increased ion sorption compared to Pb^2+^ (32 C mm^−3^) and Cd^2+^ (59 C mm^−3^) [120]. Several critical factors affecting sorption efficiency of heavy metals are presented in Figure 1.

Fiber concentration also possesses substantial effects on sorption efficiency; efficiency of ion sorption increases linearly with increasing concentrations of fiber due to concurrent production of more active sites (OH and COOH) on macromolecules. Sorption efficacy may decrease beyond the optimal sorbent dose due to increased aggregation of adsorbent molecules [120]. Pb^2+^ sorption increased for 21.78, 23.41, and 26.98 mg/L at various polymer concentrations, of 1, 2, and 3%, respectively, and at a pH of 5.0, according to Basiri and Shekarforoush [125]. Additionally, it was discovered that sorption effectiveness changes with temperature; it increases with rising temperatures before dropping after a period of time. Guiza [126] studied Cu-ion sorption from aqueous solutions by cellulose from waste orange peel. That team observed that sorption was dependent on solution pH, adsorbent dosage, contact time, metal-ion concentration, and agitation speed. According to Pal and Giri [127], the sorption efficiency of guar gum increased at up to a temperature of 40 °C and then decreased at 40 °C. The efficiency increased due to the possibility that a temperature increase would enhance the ions’ mobility and mobilize more Pb^2+^ towards the giant adsorbent molecules, which would then increase their contact with the surfaces of the adsorbents. However, the efficiency dropped beyond 40 °C due to dominant desorption of Pb^2+^ because of the increased Brownian movement [128].

### 2.4. Different Modification Technologies for Enhancing Sorption Efficiency

The ability of fibrous plants and their components, such as cellulose, hemicellulose, and lignin, to remove heavy metals from effluents has been intensively studied. Cellulose, as a plant-fiber component, works as a skeleton of natural plant-cell walls, whereas lignin and hemicellulose are distributed in the fibrous plant matrix, resulting in poor functionalities of fibers [129]. Subsequently, it is imperative to develop a suitable method for increasing the functionalities of fibers to enhance usage of plant byproducts, such as biosorbents [130]. The effectiveness of these materials can be enhanced through various types of modification techniques. Several technical approaches, including mechanical, chemical, enzymatic, and/or biological technologies, have been developed to disrupt plant cellular integrity and isolate fibers with altered structural, physicochemical, and functional characteristics to enhance sorption efficiency [68,69].

Physical modification involves altering the physical structures of fibrous plant materials, such as pore size and surface area, to increase their sorption capacities. Methods such as grinding, milling, and sieving can be used to affect physical changes. Huang and Liao [131] demonstrated that homogenization via mechanical shearing resulted in damage to the cellulose and crystallization regions of citrus peels, as well as an increase in the specific surface area and the total number of charged ions. Similarly, the molecules of cellulose and lignin were destroyed and transformed into tiny molecules during a steam explosion, increasing the sorption capacities for heavy metals [118]. Xu and Wang [132] showed that high hydrostatic pressure may significantly enhance the ability of insoluble fibers for water retention and swelling, oil holding and cation exchange, and glucose adsorption. Meanwhile, the twin-screw extrusion treatment has been shown to reduce the OHC of orange-peel fiber and increase the lead-binding ability of garlic-skin fiber [133].

Chemical modification involves treating fibrous plants with chemical reagents to introduce functional groups to their surfaces, thereby enhancing their sorption capacities. Using oxidizing compounds such as sodium chlorite and sodium periodate, for example, it is possible to introduce carboxylic and hydroxyl groups. Following a chemical treatment, Wang and Li [134] found that kiwifruit fiber treated with NaOH has greater thermal stability, but kiwifruit fiber treated with citric acid delivers higher sorption capacities for water, oil, bile acid, nitrite ions, and glucose. In this regard, Adegoke and Akinnawo [135] employed numerous surface-modification treatments, such as acid, alkaline, magnetic, and grafting modifications, for improving sorption of heavy metals, including As, Cd, Cr, Cu, Co, Fe, Hg, Mn, Ni, Pb, and Zn. They revealed that acidic treatment mostly favors the sorption process. It has been proven that some pretreatments, such as hydrochloric acid, tartaric acid, sodium carbonate, and sodium hydroxide, can effectively increase the rate of heavy-metal sorption by rice husks [136,137].

Biological modification entails treating fibrous plant materials with microorganisms or enzymes to alter their surface properties, such as charge and hydrophobicity, thereby increasing their sorption capacities. As a biological surface functionalization, cationic surfactant can be applied to remediate heavy metals in wastewater, in which case, a cationic surfactant could change the negative surface charge of a biosorbent to a positive surface charge and would have the profound ability to uptake metal anions rather than cations. Rastogi and Tiwari [138] used agroindustrial waste to synthesize a biosurfactant via submerged fermentation using *Bacillus haynesii*, and the biosurfactant could significantly remediate Pb^2+^. In addition, Dong and Du [139] implied that modified wheat straw with polyethylenimine (a highly branched molecule containing amine groups) has a paramount impact on Cu^2+^ purification from aqueous solutions. Furthermore, Chu and Zhao [140] used *Bacillus natto* to ferment millet bran. As a result of degradation of cellulose and hemicellulose by fermentation, the modified millet bran fiber developed more porous and loose structures, which increased its sorption capacity.

In conclusion, the relationship between various types of modification techniques and the respective components of the plant-fiber sorbent materials used for heavy-metal sorption is complex and dependent on the specific modification technique employed, the type of plant fiber used, and the degree of modification. The selection of an effective modification technique for fibrous plant-based sorbent materials used for heavy-metal sorption requires careful consideration of these factors.

## 3. Industrial Applications of Fibrous Plant-Based Materials and Plant-Fiber Components for Environmental Remediation of Aqueous Solutions and Wastewater

The ability of plant-fiber components, such as pectin, to bind metals is advantageous for removing heavy metals from aqueous systems, as reported in many studies [141,142]. Kumar and Kumar [143] produced ferrous ion-loaded pectin hydrogels for removal of arsenic (As) from aqueous solutions. They proposed that their compound can be used as a vehicle for water purification because of its high yield, biodegradability, and low cost [143]. Jakóbik-Kolon and Bok-Badura [144] developed calcium-crosslinked pectin (30.2% DE, degree of esterification) beads in combination with various biopolymers, karaya gum, arabic gum, and xanthan for Zn removal from water; it demonstrated the best swelling and Zn sorption at a pH of 4. In addition, Zn removal was also facilitated by physical sorption of Zn^2+^ into the complex [144]. Hastuti and Hadi [145] successfully removed ~ 44% of Pb from water using pectin derived from carrot peel at a pH of 6. Metal removal from water was accomplished using a high concentration of methoxylated nopal pectin (65% DE). After pectin treatment, more than 90% of Ca, Cu, Zn, Cr, and Ni; 67% of Pb; and 44% of Cd were eliminated by ionic contact and polar covalent bond formation [142]. Tarmizi and Ismail [146] performed another investigation on use of apple pectin (DE: 70–75%, 5 mg/L) and magnesium chloride (15 mg/L) at an alkaline pH (10). This mixture reduced the turbidity of a water supply by up to 97.71% and the iron content by 92.23% but did not significantly reduce the concentrations of other cations, such as Cd, As, Cr, and Cu. This result is most likely due to the high esterification degree of pectin, which has a limited number of accessible sites for cations. This is also attributed to the high content of Fe in the untreated sample, which favored Fe removal more than would other electrolytes [146].

In another study, Shukla and Pai [147] evaluated the potential of coir, a low-cost lignocellulosic fiber, for removal of heavy-metal ions such as Ni^2+^, Zn^2+^, and Fe^2+^ from aqueous solutions. Additionally, the fiber was chemically changed by oxidization with hydrogen peroxide before use as an adsorbent. Coir fibers were used to perform Langmuir-type adsorption. Modified coir fibers adsorbed 4.33, 7.88, and 7.49 mg/g of Ni^2+^, Zn^2+^, and Fe^2+^, respectively, compared to the 2.51, 1.83, and 2.84 mg/g by unmodified coir fibers [147]. The adsorption ability was retained only when an intermediary stage of regeneration with a diluted NaOH solution was performed following desorption. The higher metal-ion uptake in modified coir has been attributed to an ion-exchange process [147]. Notably, fibers could be regenerated with alkali and reused three times with maximum efficiency, boosting their reusability and function as a reversible ion exchanger [147]. Feng and Guo [74] demonstrated that the adsorption capacity of modified orange peel increased 4.2, 4.6, and 16.5-fold for Pb^2+^, Cd^2+^, and Ni^2+^ from wastewater, respectively, compared to that of unmodified orange peel. Furthermore, the adsorbed Pb^2+^, Cd^2+^, and Ni^2+^ ions could be recovered using a 0.05 mol/L HCl solution, and the wasted sorbent could be regenerated and reused due to immobilized behavior, which makes the biosorption process more cost-effective. Tangtubtim and Saikrasun [148] reported that alkali-treated pineapple fiber immobilized with polyethyleneimine could be used as a potential adsorbent to remove Cu^2+^ and Pb^2+^ from aqueous solutions. Hu and Huang [149] investigated Pb^2+^, Cd^2+^, Zn^2+^, and Cu^2+^ adsorption by cellulose, lignin, and hemicellulose. The results demonstrated that the highest percentage of heavy-metal removal was achieved by hemicellulose, followed by cellulose and lignin.

Pejic and Vukcevic [150] investigated the sorption capacity of short-hemp-fiber waste for Pb^2+^, Cd^2+^, and Zn^2+^ ions in aqueous media. They demonstrated that the sorption characteristics of hemp fibers improved by gradual reduction of the amount of lignin or hemicelluloses in the hemp fibers via chemical treatment. Short hemp fibers can bind metal ions (Pb^2+^, Cd^2+^, and Zn^2+^) from both single and ternary metal-ion solutions. The maximal total sorption capabilities of Pb^2+^, Cd^2+^, and Zn^2+^ ions were the same in single solutions, i.e., 0.078 mmol/g, while in ternary mixtures, they were 0.074, 0.035, and 0.035 mmol/g, respectively [150]. Mongioví and Morin-Crini [151] used plant fibers of hemp and flax in the form of felt as biosorbents to remove Al, Cd, Co, Cu, Mn, Ni, and Zn from aqueous solutions. The flax-based felt had higher biosorption capacities with respect to the studied metals than did the hemp-based felt. The highest removal efficiency was always obtained for Cu ions, and the following order of Cu > Cd > Zn > Ni > Co > Al > Mn was found for both examined biosorbents. In another study, Demirbas [152] studied adsorption of heavy-metal ions (Co^2+^ and Hg^2+^) by modified lignin from *Ailanthus altissima* wood using alkali glycerol delignification. Imran-Shaukat and Wahi [153] used various agricultural waste biomasses to adsorb metal ions for their cellulosic constituents, such as lignin, hemicellulose, lipids, extractives, sugars, proteins, and starch, which contain functional groups to participate in heavy-metal complexation. A study by Agarwal and Upadhyay [154] demonstrated that the olive stone is capable of removing Cu^2+^ from effluents.

Reshmy and Philip [155] reviewed the most practical and recent information on applying nanocellulose in heavy-metal remediation from wastewater. Faster kinetics, efficiency across a wide pH and temperature range, and low cost are the most important features of nanocellulose. Cheng and Chen [156] stated recent developments for sugarcane bagasse fiber and sugarcane-bagasse-fiber cellulose nanocrystals (SBFCNCs) as green materials in manufacturing of composites and heavy-metal sorbents. They mentioned that SBFCNCs have a high specific surface area, chemical accessibility, hydrophilic properties, and functionalization flexibility to enhance their sorption capacity towards heavy metals. Nevertheless, cellulose, pectin, starch, guar, and xanthan gums have been used for sustainable water treatment [157].

## 4. Challenges and Future Perspectives of Using Plant Fiber-Based Materials as Heavy-Metal Biosorbents

### 4.1. Effects of Process Conditions on Fibrous Plant-Based Food Waste

Recent years have witnessed a boom in biosorption-related research. It is unclear, however, whether such a substantial increase in published output has appreciably increased our understanding of the process or facilitated economic exploitation, which is so frequently the primary motivation for such studies. Most of that research focused on characterization of selected biomass types in adsorbing particular substances from solutions and the influences of physicochemical factors on biosorption. Most studies focused on metals, although a rising number also examined organic contaminants [158]. Despite the tremendous rise in creation of various biosorbents, there are still several issues related to these materials, including pH stability, sorption capacity, and durability, that must be addressed for future applications [159]. Further studies could be performed to focus on the sorption mechanism at the biosorbent–water interface.

### 4.2. Modification of Fibrous Plant-Based Food Waste and Process Intensification

It has been observed that modified fibers extracted from plant-based food yield better outcomes than unmodified fibers. Different modifications, such as physical, enzymatic, bacterial, and chemical treatments, can be used to increase fiber porosity and surface area, thereby increasing the number of sorption sites and binding functional groups on biosorbent surfaces [69]. A combination of multiple methods in the context of process intensification should be explored for increasing heavy-metal removal efficiency and possibly reducing costs.

### 4.3. Regeneration and Reusability of Fibrous Plant-Based Food Waste

Biosorption studies have been conducted for many years, but commercial use has yet to be achieved. The lack of studies on regeneration of adsorbents and their sustainable disposal is one of the major challenges to scaling up. Biosorption, on the other hand, may entail many functional groups on the surface of biomasses and is frequently nonselective, suggesting that its application to metal combinations (a frequent occurrence in waste streams) would be troublesome. As ion-exchange resins may be produced to include a single metal-binding functional group with a high affinity, they are more ideal for selective recovery of target chemicals and are more predictable for particular metal ions. The lack of selectivity and reduced resilience of plant biomass-based systems compared to ion exchange resins are frequently highlighted as key obstacles to commercialization of biosorption [160]. Suspended biomass is ineffective and unreliable in repeated, long-term applications, and its subsequent separation from treated effluents is problematic. Immobilized and/or granular biomass preparation may address the robustness and separation issues, but not the specificity issue. In addition, it should be highlighted that (bio)sorption technology moves sorbate from one medium to another, which poses concerns regarding safe disposal of loaded biosorbents, sorbate recovery, and regeneration or replacement of biosorbents [158]. The creation of particular metal-binding molecules and/or tailored highly specific fibrous plant-based biosorbents is hailed as a promising research direction; however, practical application appears to have made little progress.

### 4.4. Effects of Possible Competition between Heavy Metals on Their Sorption

Most studies were conducted using one model heavy metal at a time; however, the presence of a single heavy metal in nature, such as wastewater or polluted water, is a rare situation. For an effective ion-exchange process, it is essential to comprehend the mechanism of competitive sorption of coexisting metals on biosorbents [161]. Additionally, use of deionized water as an experimental solution for sorption of heavy-metal ions rather than the more-complex river water or wastewater is another limitation in sorption studies [162]. The effect of multiple metals in real wastewater and polluted soil on the kinetic rate of sorption can be further investigated using plant fibers.

### 4.5. Possible Practical Applications of Fibrous Plant-Based Food Waste in Water Purification

Considering the sorption capacity of fibrous plant-based food waste, use of those fibers for water remediation, such as removal of chemical residues, oil spills, and organic wastewater, could be a substantial and cost-effective technique to minimize pollution of aquifers with metal ions, marine ecosystems with oil spills, and water bodies with organic dyes. Moreover, plant-fiber components, such as the pectin derived from apples, could be used to lower turbidity of water supplies or to reduce iron and arsenic ions [146]. Therefore, further studies could be performed on sorption of Fe or As using fiber-based sorbents in the potable water of Asia, such as in Cambodia, Afghanistan, China, Japan [163], Nepal or Bangladesh [164], and India [165]; in the surface water and groundwater of Australia, Brazil, and Mexico for gold mining [163,165]; to minimize deposition of As in the sediments of natural reservoirs, such as the Haiwee Reservoir (Olancha, CA) [166]; etc.

### 4.6. Possible Practical Applications of Fibrous Plant-Based Food Waste in Remediation of Heavy-Metal-Polluted Soil

Sorption of metal ions by fibrous plant-based food waste could be an interesting option for treatment of metal-polluted soil, soil leachates, or groundwater. In a study, cocoa shells, a byproduct of the chocolate industry and rich in fibers, proteins, polyphenols, methylxanthines, etc., were used as an efficient natural adsorbent to remove Pb and other metals (Cu and Zn) from acid soil leachates [167]. The fibers of cocoa shells are mainly composed of pectin and cellulose [168]. Those results showed that around 1060–2730 mg Pb/kg could be removed from contaminated soil leachates [167]. This demonstrated that the uptake of ions in cocoa shells is dominated by ion-exchange reactions with Ca, Mg, and K ions and protons. The carboxyl and amine functional groups played a key role in the Pb uptake process. Derakhshan-Nejad and Jung [169] used raw rice husks and maple leaves for agricultural soil purification from Pb, Cu, Cd, and Zn using immobilization techniques.

Yang and Li [170] used extracts from food wastes (pineapple peel, lemon peel, grapefruit peel, and gardening crab-apple fruit) to develop a two-stage sequential washing method (extracts and/or citric acid coupled with extracts) for facile remediation of metal-contaminated agricultural soil. The removal mechanisms of Cd and Cu in soil and eluents by pineapple-peel washing agents and residues are attributed to acid activation, cation exchange, and complexation between metal ions and carboxyl groups.

## 5. Conclusions

Heavy-metal sorption is a promising approach due to its ease of use and excellent removal efficacy over a wide pH range. However, preparing suitable sorbent materials can be expensive, and some, such as commercially activated carbons, cannot be regenerated after use, making large-scale applications unsustainable. Conversion of fibrous plant-based food waste into low-cost sorbents is a renewable and ecologically benign strategy based on a “circular bioeconomy” and “green chemistry”. However, untreated plant waste can reduce sorption capacity, increase biological and chemical oxygen demand, and increase total organic carbon due to release of soluble organic carbon from plant materials. Fibrous plant-based food wastes and fibers extracted from nuts, cereals, fruits, and vegetable waste materials could be excellent sorbents for eliminating several detrimental and poisonous compounds, such as heavy metals, from wastewater and aqueous solutions. Thus, the use of fibrous plant-based food waste as biosorbents for heavy-metal remediation shows tremendous promise as a cost-effective and environmentally friendly water purification solution. To investigate biosorption technology on an industrial scale, however, several challenges, including pH stability, sorption capacity, durability, and regeneration of adsorbents, must be overcome. Further study should focus on optimizing binding capacity and process conditions to maximize efficacy. Certainly, this review article contributes to the field by providing an insight of the potential of using fibrous plant-based food waste as biosorbents for removal of heavy metals from effluents. It confirms that this waste can bind heavy metals and provides valuable insights into the factors that influence its binding capacity, such as the waste’s source, its chemical structure, and the type of metal.

## Figures and Tables

**Table 1:** Sources of toxic heavy metals, permissible limits (WHO), and their health effects.

Heavy Metal	Sources	Permissible Limit (mg/L) *	Adverse Effects on Human Health at High Concentrations	References
Arsenic (As)	Mining, coal combustion, metal smelting, phosphate fertilizers, herbicides and insecticides, semiconductor industries	0.01	Diabetes, cancer (lung, bladder, skin, liver, kidney), muscular weakness, nausea, vomiting, diarrhea, encephalopathy, neurological disorders	[9,10]
Cadmium (Cd)	Metal plating/processing, mining, battery-recycling plants, alloy industries, cigarette smoke, pigments, stabilizers	0.003	Bone and kidney damage; cancer of the skin, lungs, liver, and bladder; kidney damage; renal disorder; emphysema	[11]
Chromium (Cr)	Steel fabrication, chemical and textile industries, paints and pigments, ceramics/wood-treatment plants	0.05	Nausea and headache, liver and kidney damage, vomiting and diarrhea, skin irritation, circulatory effects, lung tumors/cancer, pulmonary fibrosis	[12]
Cobalt (Co)	Leather, jewelry, children’s toys, orthopedic and other implanted devices	0.1	Thyroid and liver damage, asthma-like allergies, heart damage, carcinogenesis	[13,14]
Copper (Cu)	Metal smelting, mining, tanneries, pigments and paints, fertilizers, cleaning, plating baths	2.5	Kidney and liver damage, Wilson’s disease, anemia, insomnia	[15]
Iron (Fe)	Cosmetics, pigments, batteries, pharmaceuticals and medical drugs	0.3	Brittle nails, constipation, depression, gastrointestinal complaints, headache, tinnitus	[16,17,18,19,20]
Lead (Pb)	Battery manufacturing, smelting industries (mining, steel, automobile, battery, paint, etc.), ceramic and glass industries, ammunition, bronze products and pipe	0.05	Kidney and brain damage, muscles (ecological balance), anemia, anorexia, circulatory- and nervous-system disease	[12,15]
Manganese (Mn)	Rocks, soil, water, steel and iron production	0.5	Motor dysfunction syndrome, Parkinson’s disease, memory loss	[21]
Mercury (Hg)	Cosmetic preparation, oil refining, paper and pulp industries, rubber processing, thermometers, batteries, paints, pharmaceuticals and medical drugs	0.001	Neurological damage, nausea, neurasthenia, fever, gastrointestinal disease (vomiting, diarrhea), paralysis, rheumatoid arthritis, blindness, anorexia	[22,23]
Nickel (Ni)	Chemical and electrochemical industries, silver refineries, stainless-steel manufacturing, electroplating, mining, paints, ink formulation units	2.0	Lung cancer, dermatitis, skin irritation, nasopharyngeal tumors, nausea, chronic asthma, coughing	[24,25]
Zinc (Zn)	Paints and pigments, pharmaceuticals, cosmetics, galvanizing, insecticides	5.0	Dehydration, anemia and increased thirst, depression, lethargy, gastrointestinal disease (vomiting, diarrhea), dizziness, skin irritation, nausea, osteoporosis, neurological signs	[24]

* The World Health Organization (WHO) has established guidelines for drinking-water quality, including limits for heavy metals and metalloids. The WHO limits for heavy metals in drinking water are typically expressed in units of milligrams per liter (mg/L) or micrograms per liter (µg/L), depending on the specific metal. The permissible limits for heavy metals in drinking water set by the WHO are generally based on average adult body weight but are designed to be protective for the general population, including children and vulnerable individuals, by incorporating safety factors to account for potential variability in individual sensitivity.

**Table 2:** Advantages and limitations of common technologies used for the removal of heavy metal from wastewater.

Technology	Mechanisms of Action	Advantages	Limitations	References
Adsorption (Commercial Activated Carbon)	Formation of Van der Waals forces Electrostatic attraction Covalent bonds Precipitation	Easy process Broad range of metal-binding capacity Suitable for a wide pH range Low cost Available Can be regenerated	Chemicals for desorption are needed Production of waste products Rapid saturation Not selective	[41] [42]
Biological Methods	Heavy metals binding to the surfaces of cells Translocation of heavy metals into cells Heavy-metal reduction	Process can be aerobic and/or anaerobic Easy process Cost-effective High efficiency High removal of biochemical oxygen demand and suspended solids A large number of species can be used in mixed or pure cultures Efficiently eliminates organic matter: NH_3_, NH_4_, iron, etc.	Favorable environment is required Complex mechanisms Slow process Low biodegradability of specific molecules Sludge foaming and bulking Microbial culture composition may change Knowledge of enzymatic processes is required	[3]
Chemical Coagulation	Coagulants form multicharged polynuclear complexes Produces quick-forming, dense, and rapid-settling flocs to remove suspended solid pollutants Coagulation occurs when particles in colloidal suspension in water/wastewater are destabilized	Cost-effective Produces sludge with good settling and dewatering characteristics Suitable for large-scale waste Easy process Mixed physicochemical process	Should be combined with other methods Large consumption of chemicals Disposal problems Low removal of arsenic pH dependency Requires adjunction of non-reusable chemicals such as coagulants or aid chemicals	[40]
Chemical Precipitation	Reaction between chemical reagents (such as iron salt, lime, and limestone) and metal ions to form insoluble precipitates	Easy process Inexpensive Most metals can be removed Adopted to high-pollutant loads	Sludge dewatering and disposal remain problems and lead to extra costs pH dependency If the metals are complexed, an oxidation step is required Ineffective in heavy-metal treatment at low concentrations	[43]
Electrocoagulation	The electric current destabilizes suspended particles and neutralizes the electric charge of the pollutants to coagulate them together	Easy process Can even settle small colloidal particles Efficiency is around three times higher than chemical coagulation Low chemical usage Pure metals can be obtained Rapid and well-controlled process Less sludge Effective for certain metal ions (such as Cu^2+^ and Cr^6+^) Suitable for medium- and small-sized communities for water remediation	High capital and a running investment Requires an expensive electrical supply and some chemicals such as salt and coagulant Anode passivation and sludge deposition on the electrode Post-treatment may be required Requires regular replacement and maintenance of electrodes Initial pH should be considered	[24] [3] [40] [42]
Fenton-Like Oxidation	The hydroxyl radicals (.OH) generated from Fenton oxidation (Fe^2+^ + H_2_O_2_) can remove heavy metals	High activity Fast reaction and rapid process Mild reaction conditions	Rusting Functions with a narrow pH range High operational costs Low water-treatment capacity Secondary pollution from additional chemicals	[41]
Flotation	A gravity-based separation process: metal-ion separation forms a liquid phase by bubble attachment	High metal selectivity High removal efficiency High overflow rates Low detention periods Production of concentrated sludge Suitable for primary cleaning Mixed physicochemical process Wide range of collectors (ionic or not ionic)	High initial capital cost High maintenance and operation costs Formation of byproducts pH dependency High energy requirement Chemicals required to control the relative hydrophobicity between particles and to reach proper froth characteristics	[40]
Ion Exchange	Reversible interchange of ions between the solid and liquid phases Ion exchange occurs between divalent metal cations (M^2+^) and functional groups (−COOH, −OH)	Fast kinetics High removal ability Selective removal of metal High quality of metal removal Easy process Can be applied to both continuous and batch flow Can be combined with other techniques, such as precipitation and filtration	Only appropriate for low concentrations Highly sensitive to pH Adsorbents require regeneration or disposal Secondary pollution Synthetic resins are expensive Fouling on ion exchange media Low binding affinity Rapid saturation and clogging of reactors Saturation of the cationic exchanger before ionic resin Beads easily fouled by particulates and organic matter; requires physicochemical pretreatment (carbon adsorption and sand filtration) to remove contaminants	[34]
Membrane Filtration	Based on the particle sizes of the pores of the membranes and the size of the heavy metal to be removed	Space-saving Less sludge production Requires a lower amount of chemicals Wide range of membranes Simple and rapid process	High energy consumption Membrane restoration is required High investment cost Less output Membrane is application-dependent Not effective at low feed concentrations	[40] [44]
Photocatalysis	An oxidation process Having strong oxidizing power, photocatalysts can destroy heavy-metal complexes and free them from the metal ions, and are simultaneously capable of oxidizing and degrading organic complexes	Waste is less harmful Removes metals and organic pollutants simultaneously Little or no consumption of chemicals Rapid degradation Pollutant mineralization	Still limited to laboratory scale Long duration Technical constraints Economically unfriendly Byproduct formation Less output	[24]

**Table 3:** Commonly used fibrous plant-based food waste for heavy-metal removal from wastewater and aqueous solutions.

Sorbents	Heavy Metals Removed	Sorption Conditions	Modification Method	Mechanisms of Action	Sorption Capacity (mg/g)	References
*Artocarpus nobilis* (Peel)	Ni^2+^	pH of 4 90 min 175 °C	HNO_3_	Ion exchange	Ni^2+^: 0.012	[107]
Black Oak (Bark)	Hg^2+^	pH of 2–10 20–150 min Adsorption dose of 20–60 mg/L	None	Complexation, adsorption on surface, diffusion, and ion exchange	Hg^2+^: 400	[81]
Coconut (Shell)	Cd^2+^, Pb^2+^	pH of 2–10 20–150 min Adsorption dose of 20–60 mg/L	None	Complexation, adsorption on surface, diffusion, and ion exchange	Cd^2+^: 285 Pb^2+^: 263	[81]
Cantaloupe (Peel)	Cd^2+^, Cu^2+^, Pb^2+^	pH of 5–7	Acrylic acid	Ion exchange and complexation	Cd^2+^: 45.4 Cu^2+^: 33.1 Pb^2+^: 143.3	[108]
Carrot (Residue)	Cr^3+^, Cu^2+^, Zn^2+^	pH of 4 Initial ion concentration of 20 to 500 mg/L	None	Ion exchange	Cr^3+^: 1.65 Cu^2+^: 1.82 Zn^2+^: 1.45	[78]
Lemon (Peel)	Cd^2+^	pH of 5 45 min Initial ion concentration of 45 mg/L Particle size of 0.24–0.42 mm	Protonation and HNO_3_	Ion exchange	Cd^2+^: 32.5	[109]
Potato (Peel)	Cu^2+^	pH of 6 20 min 30 °C Initial ion concentration of 150 mg/L Particle size of 0.2 mm	None	Surface complexation and ion exchange	Cu^2+^: 0.15	[110]
Potato (Shell)	Cu^2+^, Cd^2+^	pH of 6.8 200 min (Cd) 50 min (Cu)	None	Electrostatic interaction	Cd^2+^: 90 Cu^2+^: 41.7	[80]
Soybean (Straw)	Cu^2+^	pH of 6 60 min	Citric acid	Ion exchange	Cu^2+^: 48.2–48.8	[111]
Sunflower (Stalk and Leaves)	Cd^2+^, Cu^2+^	pH of 6 120 min (Cd) 50 min (Cu)	None	Electrostatic interaction	Cd^2+^: 63.3 Cu^2+^: 30.3	[80]
Tangerine (Peel)	Cd^2+^, Co^2+,^ Cr^3+^, Cu^2+^, Mn^2+^, Ni^2+^, Pb^2+^, Zn^2+^	pH of 5 20 min Room temperature Adsorbent dose of 1–4 g/L	Nitric acid	Ion exchange	Cd^2+^: 0.003 Co^2+^: 0.01 Cr^3+^: 0.01 Cu^2+^: 0.002 Mn^2+^: 0.01 Ni^2+^: 0.01 Pb^2+^: 0.002 Zn^2+^: 0.003	[61]
Wheat (Bran)	Cr^6+^	pH of 2–10 20–150 min Adsorption dose of 20–60 mg/L	None	Complexation, adsorption on surface, diffusion, and ion exchange	Cr^6+^: 310	[81]
Wheat (Bran)	As^3+^, Cd^2+^, Hg^2+^, Pb^2+^	pH of 7 37 ℃	None	Ion exchange	As^3+^: 0.98 Cd^2+^: 36.1 Hg^2+^: 39.6 Pb^2+^: 58.2	[112]
